# Quinine Amaurosis—Report of Two Additional Cases

**Published:** 1911-05

**Authors:** B. H. Booth

**Affiliations:** Drew, Miss.


For Texas Medical Journal.

### Quinine Amaurosis—Report of Two Additional Cases.

BY B. H. BOOTH, M. D., DREW, MISS.

In the August issue I reported three case of quinine amaurosis, since which time I have seen two other cases, which I desire to report.

CASE 1.—Baby, F—, negro, male, age 15 months. When I saw him he had had three chills, about twenty-two hours apart. He had been given nothing except three or four tablespoonfuls of castor oil and various and sundry teas. His parents said that he would not get entirely clear of fever before another chill would come on. I started giving him calomel and soda by the mouth, and as it was then nearly sixteen hours since the last chill and only about six hours till the next one was due, I gave him fifteen grains of quinine and urea hydrochloride hypodermically, deep into the buttocks. On visiting him the next morning, I found that he had missed the chill but that he was completely blind. I gave him no more quinine, protected his eyes from the light and kept them cleansed with saturated solution of boracic acid, and in four days eyesight began to return and was complete a week from time of commencement.

CASE 2.—B. McK—, white, male, age 14 months. Patient was suffering with a severe cold and inflammation of the middle ear (right side), temperature 101, pulse 98, respiration 25; gave no history of a chill but liver and spleen were greatly enlarged. Gave him a course of calomel, soda and blue powders to be followed by castor oil and then syrup of quinine to be given. The next day patient was felling better; temperature 100, pulse 90, respiration 21. I ordered the quinine kept up all day and night. On the day following at 8 a. m. he was clear of fever, but they had failed to give him the quinine as directed, and at 10 a. m. he had a "congestive chill," the temperature reaching 105. I reduced the fever with cold water and started him on another course of calomel and soda and gave him ten grains of quinine and urea hydrochloride hypodermically and repeated in twelve hours. I then lanced the abscess, which discharged freely, and from that time the ear gave him no trouble whatever. After these two hypodermics of ten grains each he had no further chills and no more quinine was administered. About twenty-four hours from the time of administering the first hypodermic there was a beginning amaurosis, which within twenty-four hours was complete. He was placed on tincture nux vomica and potassium iodide. The eyes were protected from light and kept cleansed with saturated solution of boracic acid. There was total blindness for about six weeks, but from that time on there was slow return of vision for about two weeks, and three weeks from beginning return of sight there was full return of sight with nothing about the eyes to indicate that they had ever been blind.

